# StSN2 interacts with the brassinosteroid signaling suppressor StBIN2 to maintain tuber dormancy

**DOI:** 10.1093/hr/uhad228

**Published:** 2023-11-08

**Authors:** Shifeng Liu, Chengcheng Cai, Luopin Li, He Wen, Jie Liu, Liqin Li, Qiang Wang, Xiyao Wang

**Affiliations:** State Key Laboratory of Crop Gene Exploration and Utilization in Southwest China, Sichuan Agricultural University, Chengdu 611130, China; State Key Laboratory of Crop Gene Exploration and Utilization in Southwest China, Sichuan Agricultural University, Chengdu 611130, China; State Key Laboratory of Crop Gene Exploration and Utilization in Southwest China, Sichuan Agricultural University, Chengdu 611130, China; State Key Laboratory of Crop Gene Exploration and Utilization in Southwest China, Sichuan Agricultural University, Chengdu 611130, China; State Key Laboratory of Crop Gene Exploration and Utilization in Southwest China, Sichuan Agricultural University, Chengdu 611130, China; State Key Laboratory of Crop Gene Exploration and Utilization in Southwest China, Sichuan Agricultural University, Chengdu 611130, China; State Key Laboratory of Crop Gene Exploration and Utilization in Southwest China, Sichuan Agricultural University, Chengdu 611130, China; State Key Laboratory of Crop Gene Exploration and Utilization in Southwest China, Sichuan Agricultural University, Chengdu 611130, China

## Abstract

After harvest, potato tubers undergo an important period of dormancy, which significantly impacts potato quality and seed vigor. *StSN2* has been reported as a key gene for maintaining tuber dormancy; in this study, we explored the molecular mechanism by which *StSN2* maintains dormancy. StBIN2 was first identified as a candidate protein that interacts with StSN2 by co-immunoprecipitation/mass spectrometry, and both qPCR and enzyme activity experiments showed that *StSN2* can promote the StBIN2 expression and activity. In addition, the interaction between StSN2 and StBIN2 was verified by yeast two-hybrid, luciferase complementation experiments and co-immunoprecipitation. Bioinformatics analysis and site-directed mutagenesis confirmed the critical role of cysteine residues of StBIN2 in its binding to StSN2. Similar to that of *StSN2*, overexpression of *StBIN2* extended the dormancy of potato tuber. Interaction between StSN2 and StBIN2 increased the activity of the StBIN2 enzyme, inhibited the expression of *StBZR1*, and suppressed BR signaling. On the contrary, this interaction promoted the expression of *StSnRK2.2/2.3*/*2.4*/*2.6* and *StABI5*, key genes of ABA signaling, and the phosphorylation of *StSnRK2.3*, thereby promoting ABA signaling. Altogether, our results indicate that StSN2 interacts with StBIN2 through key cysteine residues and StBIN2 maintains tuber dormancy by affecting ABA and BR signaling. Findings of this research offer new insights into the molecular mechanism by which StSN2 maintains potato tuber dormancy through interaction with StSIN2 and provide guidance for potato improvement.

## Introduction

Potato (*Solanum tuberosum* L.) is a tuber crop that constitutes the fourth largest food crop worldwide, behind only corn, rice, and wheat, and China is the largest potato producer in the world [1, 2]. After potato harvest, a period of dormancy is required for potato tubers to begin sprouting. Tuber dormancy is a complex physiological process that developed as an adaptation strategy to cope with stressful environments [3]. The match between tuber dormancy time and storage, planting, and shelf periods poses a significant challenge to dormancy control, and improper sprouting is a constant concern for the potato industry [4]. Recent studies have found several key genes that play roles in the maintenance of dormancy. *DOG1* is a key factor that promotes plant seed dormancy, and studies have shown that it may maintain dormancy by affecting abscisic acid (ABA) levels in Arabidopsis seeds [5]. ABA is the only plant hormone known to function in the maintenance of seed dormancy, and studies have demonstrated that genes involved in signal transduction, such as *ABI1*, *ABI3*, and *SnRK2s*, play crucial roles in promoting seed dormancy [6]. The simultaneous mutation of *SnRK2.2*/*2.3*/*2.6* in Arabidopsis induced a range of detrimental effects during seed development. Specifically, this mutation results in the loss of seed dormancy and a concomitant increase in ABA content within seeds [7]. Brassinosteroid (BR) has an antagonistic effect on ABA in regulating seed dormancy [8]. ABA promotes the expression of *BIN2*, the only known negative regulatory factor of BR, through *ABI1*, *ABI2*, and *ABI5*, which affects BR synthesis and maintains seed dormancy [9]. In potato, silencing of the amylase *StAmy23* leads to prolonged dormancy [10]. The dormancy period of the tubers overexpressing the *PPase* gene was also found to be shortened, while the dormancy period of tubers was prolonged when the *PPase* gene was silenced, indicating that *PPase* negatively regulates the dormancy of potato tubers [11]. The underlying molecular mechanisms of potato dormancy are highly complex, involving multiple genes and proteins. Therefore, in-depth research into the regulatory mechanism of tuber dormancy, especially the identification of key genes, is particularly important.

The Snakin/GASA family is widely distributed in plants and plays a vital regulatory role in several plant growth and development processes, such as seed germination, lateral root formation, stem elongation, flowering and fruit development, biotic and abiotic stress response, and hormone signaling [12]. Snakin-2 (StSN2) belongs to the Snakin/GASA protein family, which was the first reported antimicrobial peptide in potato, and its expression was enhanced by gibberellin (GA3) treatment [13], suggesting a potential link between its function and hormone signaling [14]. Our prior research indicated a positive correlation between *StSN2* expression and dormancy, and overexpression of *StSN2* was found to considerably prolong the dormancy period of tubers [15]. Further investigation revealed that *StSN2* significantly diminishes the accumulation of lignin precursors in the periderm, thus delaying skin cracking and dehydration and maintaining tuber dormancy [16]. In addition, StSN2 interacts with glyceraldehyde-3-phosphate dehydrogenase (GAPC) to enhance its activity and suppress bud growth [17]. Although research on the maintenance of dormancy through *StSN2* has made some progress, the molecular mechanism by which *StSN2* interacts with hormones to maintain dormancy remains unclear.

Brassinosteroid-insensitive 2 (BIN2), a well-studied member of the glycogen synthase kinase 3 (GSK3) family, functions as a negative regulator of BR signal transduction and plays a crucial role in signaling processes [18]. As a kinase, BIN2 regulates the activity, stability, and subcellular localization of various proteins by phosphorylation and dephosphorylation [19]. In Arabidopsis, there are at least 10 GSK3 family members, and BIN2 has been shown to interact with ABI1 and ABI2. ABI1 and ABI2 are members of the PP2C family and are negative regulators of ABA signaling. BIN2 is the hub of the crosstalk between ABA and BR signaling. ABA promotes BIN2 phosphorylation by inhibiting the activity of ABI2 through ABA receptors and BIN2 interacts with and phosphorylates SnRK2s to activate their kinase activities. The PP2Cs-SnRK2s-BIN2 complex controls responses to abiotic stresses, such as drought, highlighting the synergy between BR and ABA [20]. In addition, BIN2 has been shown to positively regulate ABA signaling in Arabidopsis by phosphorylating SnRK2.2/2.3 [21]. Furthermore, BIN2 regulates the development of plant stomata by inhibiting the activity of MAPKKK through phosphorylation [22]. By modulating the interplay between ABA and BR hormones, BIN2 regulates the germination and subsequent development of plant seeds.

In this study, we screened the candidate interacting proteins of StSN2 by co-immunoprecipitation/MS and identified and identified StBIN2. *StBIN2* and *StSN2* exhibit correlated expression trends during storage, and overexpression of *StSN2* increases the expression level and enzymatic activity of StBIN2. Moreover, interaction between the StSN2 and StBIN2 proteins was confirmed through yeast two-hybrid, Co-IP and luciferase complementation experiments. StSN2 binds to specific cysteine residues within StBIN2 to increase its enzymatic activity. In addition, we found that the interaction between these proteins maintained tuber dormancy by promoting ABA signaling and inhibiting BR signaling. Our research shows that the StSN2-StBIN2 module maintains tuber dormancy by affecting the ABA and BR signaling pathways.

## Results

### *StSN2* enhances *StBIN2* expression

**Figure 1:**
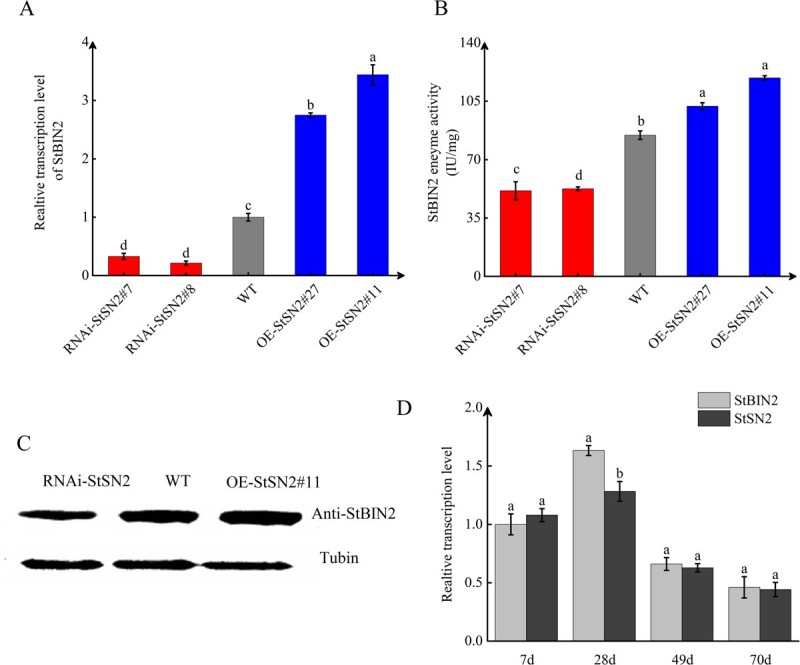
*StSN2* enhances StBIN2 expression in potato tuber. **A** Relative transcript level of *StBIN2* in the WT, RNAi, and overexpression lines detected by qRT-PCR. Data were normalized using the 2^−ΔΔCt^ method and *elongation factor 1α* (*EF-1α*) was used as the internal reference for data normalization. The transcript level of *StBIN2* in the WT control was sent to 1*.***B** Western blotting assay. *β-tubulin* was used as the internal reference. **C** Determination of StBIN2 enzyme activity. **D** Relative transcript levels of *StSN2* and *StBIN2* during tuber storage. The *x*-axis displays days of tuber storage at 20°C and the *y*-axis displays the transcript levels of *StSN2* and *StBIN2*. EF-Iα served as the internal reference for data normalization. The transcript level of *StBIN2* at 7 days was sent to 1. Data are shown as means ± SD (*n* = 3, Student’s *t*-test). Error bars represent standard deviation of three replicates. Different lowercase letters indicate significant differences (*P* ≤ 0.05).

Previous studies have demonstrated the crucial role of *StSN2* in maintaining the dormancy of tubers. To investigate the underlying molecular mechanisms, transcriptome and proteome analyses were conducted on transgenic tubers with either enhanced or reduced *StSN2* expression levels [15]*.* This analysis revealed that *StSN2* influences the expression of the BR signaling pathway gene *StBIN2*. To study the relationship between *StSN2* and *StBIN2*, we silenced and overexpressed *StSN2* in ‘Chuanyu 10’ and generated 23 RNAi lines and 27 overexpression lines. RNAi lines 7 and 8 and overexpression lines 11 and 27 were randomly selected for subsequent experiments (Supplementary Fig. S1) [16]. The qPCR results showed that compared with the WT control, *StBIN2* transcript level in *OE-StSN2#27* and *OE-StSN2#11* was 2.67- and 3.33-fold, whereas that in the two RNAi lines was 0.36- and 0.42-fold lower (Fig. 1A). In keeping with the qRT-PCR results, western blotting assays showed that the abundance of StBIN2 protein in *OE-StSN2#11* and *RNAi-StSN2#8* was 2-fold higher and 0.5-fold lower, respectively, compared with the WT control (Fig. 1B). Furthermore, StBIN2 enzyme activity was 1.46- and 1.76-fold higher in *StSN2* overexpression lines and 0.65- and 0.63-fold lower in the RNAi lines than in the WT control (Fig. 1C). The above results suggest that *StSN2* modulates the expression and enzyme activity of StBIN2 in potato. In order to confirm the correlation between*StBIN2* and *StSN2* during tuber dormancy, we stored harvested tubers and measured the transcript levels of *StSN2* and *StBIN2* during storage. The qRT-PCR results showed that StSN2 and StBIN2 transcript levels first increased and then rapidly decreased during the entire storage period until dormancy was terminated. Compared with storage at 28 days, the transcript level of *StSN2* and *StBIN2* was decreased by 2.47- and 3.54-fold, and 2.03- and 2.9-fold, respectively, after stored for 49 days and 70 days (Fig. 1D).

### StSN2 physically interacts with StBIN2

**Figure 2:**
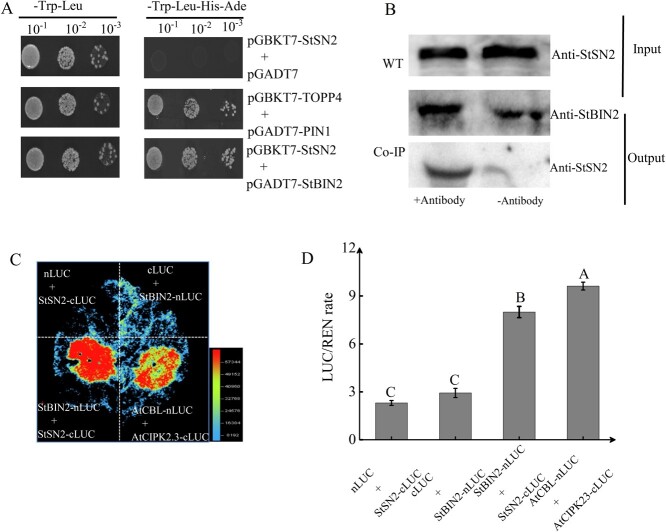
StSN2 interacts with StBIN2. **A** The interaction between StSN2 and StBIN2 examined by yeast two-hybrid assays. TOPP4 and PIN1 were used as positive controls, and AD-empty and StSN2-BD were used as the negative control. **B** The interaction between StSN2 and StBIN2 detected by protein immunoprecipitation assay. **C** The interaction between StSN2 and StBIN2 detected by luciferase complementation assay. AtCBL1 and AtCIPK23 were used as positive controls, and StSN2-cLUC/nLUC and cLUC/StBIN2-nLUC were used as negative control. **D** Dual luciferase activity assay in *Nicotiana benthamiana* leaves. Luciferase (LUC) activity was normalized to that of Renilla luciferase (REN). Data are shown as means ± SD (*n* = 3, Student’s *t*-test). Error bars represent the standard deviation of three replications. Different capital letters indicate significant differences (*P* ≤ 0.01).

To elucidate the molecular mechanism by which *StSN2* maintains the dormancy of potato tuber, we screened for interacting proteins of StSN2 through co-immunoprecipitation/mass spectrometry (Co-IP/MS) experiments. This analysis identified StBIN2 as a potential interacting protein (Supplementary Table S1). The interaction between StSN2 and StBIN2 was confirmed by yeast two-hybrid (Fig. 2A), Co-IP (Fig. 2B), and luciferase complementation assays (Fig. 2C). The results of Y2H demonstrate that as the dilution gradient increased, both StSN2 + StBIN2 and the positive control (TOPP4 + TOP1) were able to grow on quadruple dropout medium (QDO). However, StSN2 + AD-empty was unable to grow under the same condition. In the Co-IP analysis, both the StSN2 and StBIN2 proteins could be detected by corresponding antibodies in the input. Specifically, after addition of the StBIN2 antibody, StSN2 was pulled down and could be detected. By contrast, the StSN2 protein could not be detected without the presence of the StBIN2 antibody. Consistent with this finding, fluorescence was detected around *Nicotiana benthamiana* leaves injected with StSN2 + StBIN2 and the positive control (AtCBL+AtCPK23), whereas no fluorescence was detected around the negative controls StSN2-cLUC+nLUC and cLUC+StBIN2-nLUC (Fig. 2D). Results of these analyses provide solid evidence for an interaction between the StSN2 and StBIN2 proteins.

### StSN2 enhances StBIN2 enzyme activity

**Figure 3:**
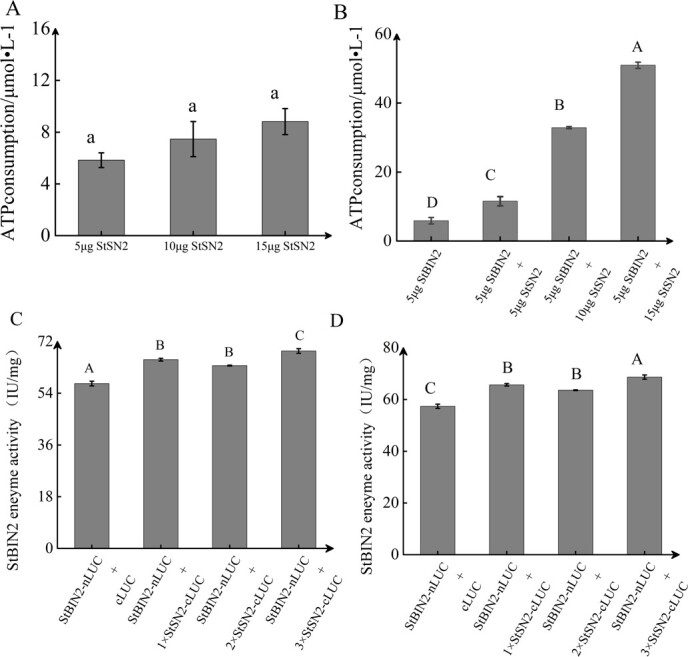
StSN2 enhances StBIN2 enzyme activity. **A** The amount of ATP consumed by different amounts of StSN2 protein. **B** The amount of ATP consumed by 5 μg StBIN2 alone or a combination of 5 μg StBIN2 with different mass of the StSN2 protein. **C** Dual luciferase activity assay by 1× StBIN2 bacterial solution alone or a combination of 1× StBIN2 bacterial solution and different amounts of the StSN2 bacterial solution. Luciferase (LUC) activity was normalized to that of Renilla luciferase (REN). **D** Determination of StBIN2 enzyme activity by enzyme-linked immunosorbent assay (ELISA). Data are shown as means ± SD (*n* = 3, Student’s *t*-test). Error bars represent standard deviation of three replicates. Different capital letters indicate significant differences (*P* ≤ 0.01) and different lowercase letters indicate significant differences (*P* ≤ 0.05).

To confirm whether StBIN2 activity was increased in *StSN2* overexpression lines due to direct interaction with StSN2, His-tagged StBIN2 and StSN2 proteins were expressed in the BL21 strain of *Escherichia coli* and purified. Protein kinases possess distinctive phosphorylation profiles that serve as indicators of enzyme activity, reflecting the consumption of ATP. First, to exclude effect of the StSN2 protein, we tested ATP consumption by StSN2 at different mass levels and found that ATP consumption did not significantly increase when more StSN2 protein was added (Fig. 3A). Subsequently, we combined the StBIN2 and StSN2 proteins in varying mass ratios and compared the ATP consumption to that of StBIN2 alone and found that StBIN2 activity with 5 μg StBIN2 + 15 μg StSN2 was 10-fold higher than that of the control (Fig. 3B). These results indicate that the interaction of the two proteins enhances StBIN2 activity. To confirm the above findings in planta, we transiently co-expressed StSN2-cLUC and StBIN2-nLUC in *Nicotiana benthamiana* leaves. The interaction intensity of StSN2 and StSN2 increased gradually with increase in the proportion of StSN2 (Supplementary Fig. S2); this was accompanied by increased luciferase activity (Fig. 3C). The enzymatic activity of StBIN2 in the presence of StSN2 was significantly higher compared with the control in which StSN2 was absent (Fig. 3D). Taken together, these results support the notion that StSN2 interacts in planta with StBIN2 to enhance its activity.

### StSN2 binds to specific cysteine residues in StBIN2

**Figure 4:**
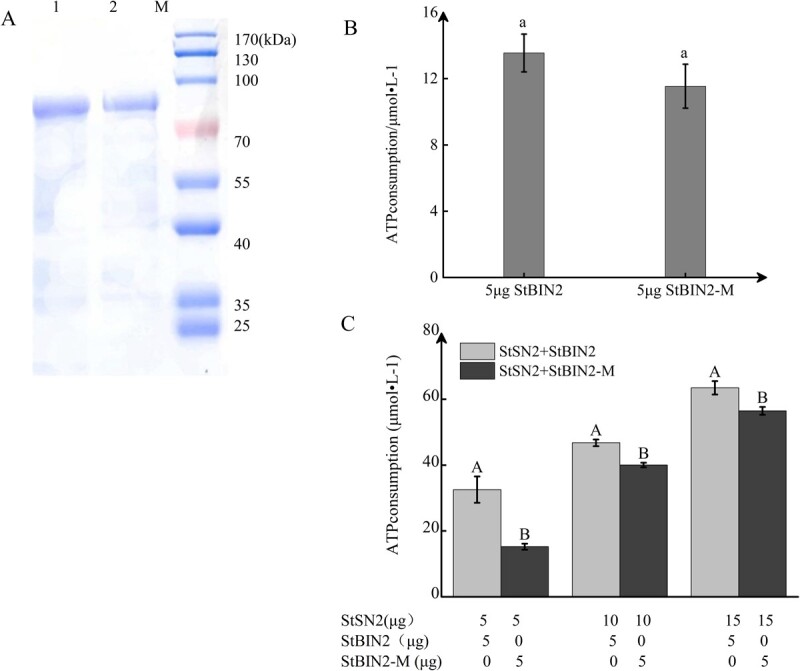
StSN2 and StBIN2 interact via cysteine residues in StBIN2. **A** Coomassie brilliant blue staining of StBIN2 and StBIN2-M proteins. Lane 1, StBIN2 protein; lane 2, StBIN2-M protein; M, protein marker. **B** Comparison of the amount of ATP consumed by StBIN2 and StBIN2-M. **C** Consumption of ATP by StSN2 + StBIN2/StBIN2-M proteins. Data are shown as means ± SD (*n* = 3, Student’s *t*-test). Error bars represent the standard deviation of three replicates. Different capital letters indicate significant differences (*P* ≤ 0.01) and different lowercase letters indicate significant differences (*P* ≤ 0.05).

The above results indicated that StSN2 interacts with StBIN2 to enhance its activity. In order to explore the mechanism underlying the interaction between StSN2 and StBIN2, we predicted their binding sites using the yeast two-hybrid antibody optimization system (Y2H-AOS) method [32]. The results of this analysis suggest that StSN2 and StBIN2 likely bind to each other through multiple binding sites, with cysteines being the most frequently predicted interaction points (Supplementary Fig. S3). BIN2 has 9 residues that are capable of forming disulfide bonds, and StSN2 was predicted to bind to these cysteine sites to enhance StBIN2 activity. Disulfide bonds serve as covalent cross-linking regulatory switches that control the activity and denaturation of enzymes under oxidative-reductive conditions [34]. A gene synthesis approach was employed to replace all cysteine residues with alanine residues at positions 60, 100, 162, 183, 202, 229, 267, 313, and 319 of the StBIN2 protein. Therefore, the mutated StBIN2 was designated StBIN2-M (Supplementary Fig. S4) and the protein was expressed in the BL21 strain of *E. coli* and purified*.* SDS-PAGE electrophoresis and Coomassie brilliant blue staining detected a single target protein (Fig. 4A). We then assessed and compared the enzyme activities of StBIN2 and StBIN2-M based on the amount of ATP consumed and found no significant difference (Fig. 4B). However, when co-incubated with StSN2, the amount of ATP consumed by StBIN2-M was reduced by 63%, 14%, and 12% compared with that by StBIN2 with the StSN2/StBIN2 (or StSN2/StBIN2-M) combination of 5 μg StBIN2 + 5 μg StSN2, 5 μg StBIN2 + 10 μg StSN2, and 5 μg StBIN2 + 15 μg StSN2, respectively. This result indicates that the binding of StBIN2 to StSN2 is weakened as a result of the cysteine-to-alanine substitutions, leading to reduced StBIN2 activity. However, despite the loss of these cysteine residues, the amount of ATP consumed continued to increase with increased levels of StBIN2-M, suggesting that the interaction between StBIN2 and StSN2 was still taking place.

### StBIN2 overexpression delays tuber sprouting

**Figure 5:**
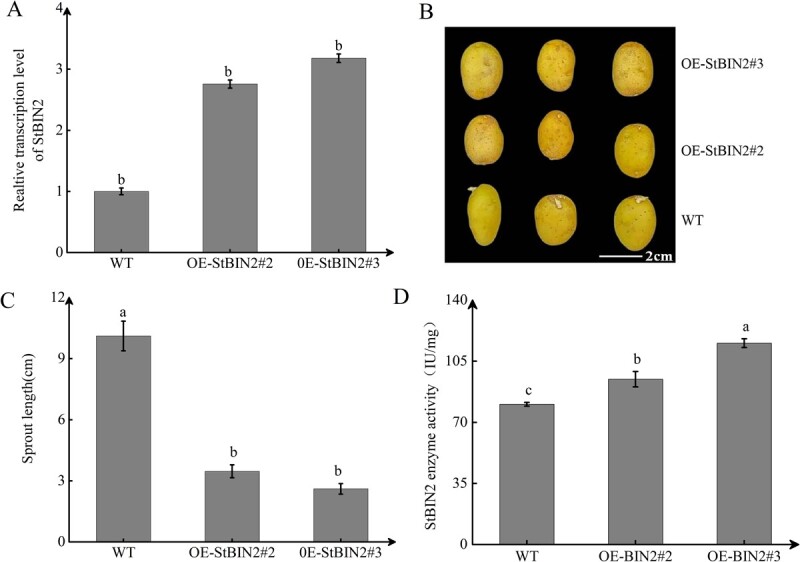
Overexpression of *StBIN2* maintains tuber dormancy. **A** qRT-PCR detection of *StBIN2* in the WT and the two *StBIN2* overexpression lines. Data were normalized using the 2^−ΔΔCt^ method and *EF-1α* was used as the internal reference for data normalization. The transcript level of *StBIN2* in the WT control was sent to 1. **B** Tuber sprouting of the WT and *StBIN2* overexpression lines. **C** Comparison of sprout length among the WT and two *StBIN2* overexpression lines. **D** Determination of StBIN2 enzyme activity in the WT and the two *StBIN2* overexpression lines by ELISA. Tubers were stored at 20°C in the dark for 60 days prior to being analyzed. Data are shown as means ± SD (*n* = 3, Student’s *t*-test). Error bars represent the standard deviation of three replications. Different capital letters indicate significant differences (*P* ≤ 0.01) and different lowercase letters indicate significant differences (*P* ≤ 0.05).

To further investigate the function of StBIN2 in maintaining tuber dormancy, we obtained StBIN2 overexpression lines through genetic transformation. qRT-PCR analysis showed that *StBIN2* transcript level in *OE-StBIN2#2* and *OE-StBIN2#3* was 2.75- and 3.18-fold of that in the WT (Fig. 5A). After 60 days of storage, we observed and measured the length of sprouts and found that StBIN2 overexpression significantly extended tuber dormancy (Fig. 5B), resulting in slow sprout growth. Specifically, sprout length of the WT was 3- and 3.4-fold greater than that of *OE-StBIN2#2* and *OE-StBIN2#3*, respectively (Fig. 5C); by contrast, no significant difference in sprout thickness was detected (Supplementary Fig. S5). In addition, StBIN2 activity in *StBIN2#2* and *OE-StBIN2#3* was 1.8- and 2.5-fold higher than that of WT (Fig. 5D). Taken together, these results indicate that expression of *StBIN2* leads to increased StBIN2 enzyme activity and prolonged tuber dormancy.

### StBIN2 act as important modulators in the ABA/BR signaling pathway

**Figure 6:**
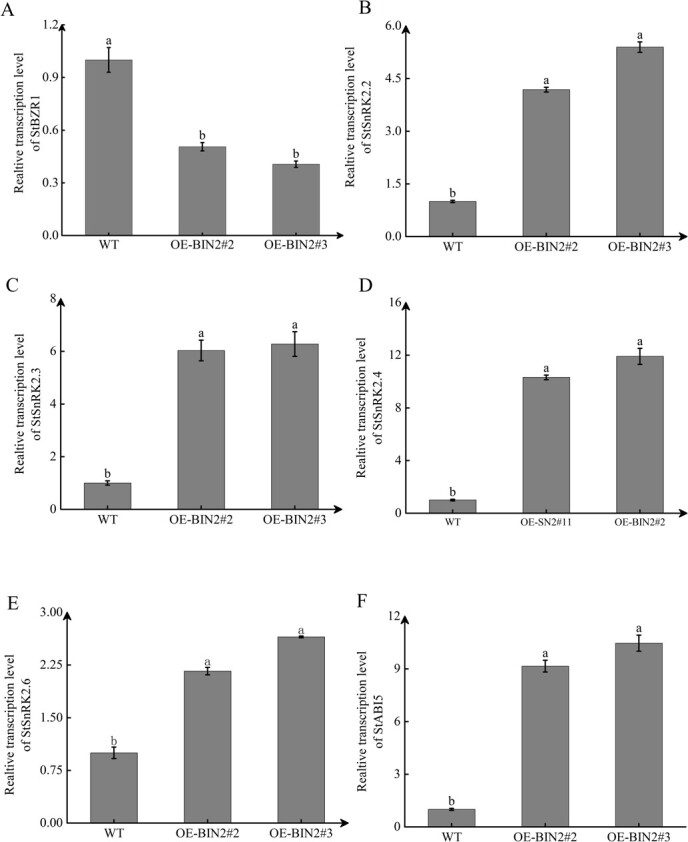
Transcript levels of key genes in BR and ABA signaling in the tuber of WT, *StBIN2* overexpression lines during storage. **A**–**F** The transcription levels of *StBZR1*, *StSnRK2.2*, *StSnRK2.3*, *StSnRK2.4*, *StSnRK2.6*, and *StABI5* in *StBIN2* overexpression lines relative to the WT. Data were normalized using the 2^−ΔΔCt^ method and EF-1α was used as the internal reference for data normalization. The transcript level of each target gene in the WT was set to 1. Data are shown as means ± SD (*n* = 3, Student’s *t*-test). Error bars represent the standard deviation of three replicates. Different lowercase letters indicate significant differences (*P* ≤ 0.05).

BIN2 is known to be a negative regulator of BR signaling [35]. Recent studies have also shown that BIN2 interacts with SnRK2s to activate ABA signal transduction through phosphorylation [21]. To further understand the function of *StBIN2* in ABA/BR signaling, we measured the transcript levels of key genes involved in ABA and BR signaling in *OE-StBIN2#2*, *OE-StBIN2#3*, and WT tuber budding eyes that had been stored for 30 days. qRT-PCR analysis showed that the transcription level of *StBZR1*, a key transcription factor that positively regulates BR signaling by activating downstream gene expression, in *StBIN2* overexpression lines was 0.2- and 0.25-fold of the WT (Fig. 6A), suggesting that StBIN2 can suppress BR signaling. The transcript level of key ABA signaling genes, including *StSnRK2.2/2.3/2.4/2.6*, which activates ABA signaling through phosphorylation (Fig. 6B–E), and *ABI5* (Fig. 6F), which is an important downstream transcription factor in ABA signaling, was increased in *OE-StBIN2#2* and *OE-StBIN2#3* compared with the WT, suggesting that StBIN2 positively regulate ABA signaling. Thus, it is reasonable to believe that *StBIN2* plays a critical role in ABA/BR signaling during tuber dormancy.

### StBIN2 phosphorylates StSnRK2.3 to enhance ABA signaling

**Figure 7:**
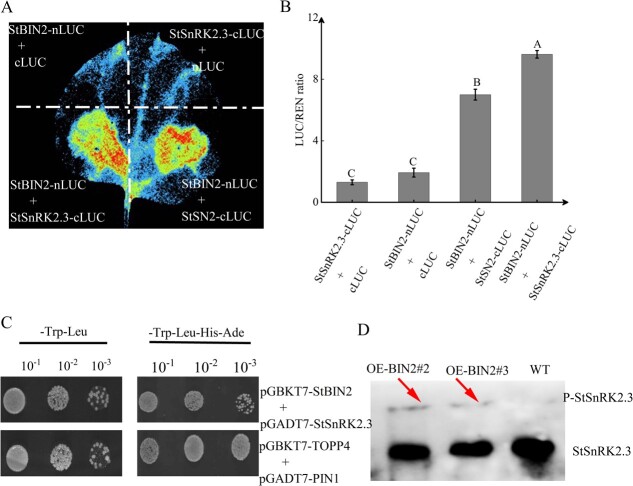
StBIN2 interacts with and phosphorylates StSnRK2.3. **A** Luciferase complementation assay of StSnRK2.3 and StBIN2. **B** Dual luciferase activity assay of StSnRK2.3 and StBIN2. Data are shown as means ± SD (*n* = 3, Student’s *t*-test). Error bars represent the standard deviation of the three replicates. Different capital letters indicate significant differences (*P* ≤ 0.01). **C** Yeast two-hybrid assay of StSnRK2.3 and StBIN2. TOPP4 and PIN1 were used as positive controls. **D** Chemiluminescence detection of phosphorylated proteins on PVDF membranes using the StSnRK2.3 antibody.

To understand the specific molecular mechanism by which StBIN2 regulates ABA signaling and investigate whether StBIN2 interacts with SnRK2.3, which is a positive regulator of ABA signaling [36], we analyzed the expression of StSnRK2.3 in potato tubers during storage and performed luciferase complementation and yeast two-hybrid and assays. Fluorescence was detected around *N. benthamiana* leaves injected with StSnRK2.3 + StBIN2 (Fig. 7A and B), suggesting that StBIN2 indeed interacts with StSnRK2.3 *in vitro*. The Y2H assay further confirmed this specific interaction (Fig. 7C). We then performed phos-tag mobility shift assays on total proteins extracted from tuber budding eyes that had been stored for 30 days and detected a slow-migrating form of StSnRK2.3 that corresponded to the phosphorylated StSnRK2.3 protein in the two *StBIN2* overexpression lines, whereas this band is absent from the WT potato tuber (Fig. 7D). This result provides strong evidence that StBIN2 phosphorylates SnRK2.3 *in vivo.* Considering the high level of similarity of SnRK2 members, it is likely that StBIN2 also phosphorylates other SnRK2s proteins, although this requires further investigation.

**Figure 8:**
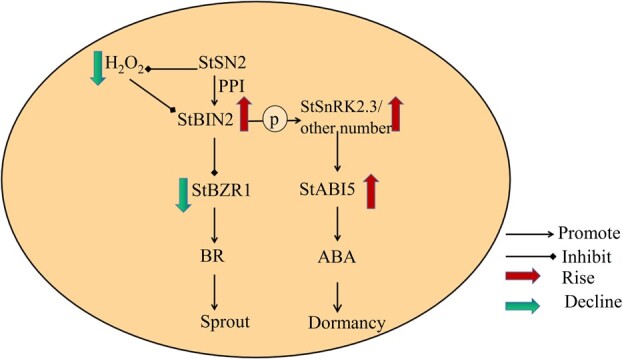
Working model depicting the regulatory mechanism of StSN2 and StBIN2 in maintaining tuber dormancy.

## Discussion

Potato tuber dormancy is a complex physiological process that is directly or indirectly affected by numerous environmental, physiological, and genetic factors [37]. At present, the molecular mechanisms of potato tuber dormancy are poorly understood, which severely limits the development of the potato industry. In our previous studies, we found that *StSN2* overexpression extends tuber dormancy, whereas reduced *StSN2* expression accelerates sprouting [16]. In addition, the expression of *StSN2* was also positively correlated with the degree of dormancy across multiple different potato varieties [17]. Proteomics and transcriptomics analysis showed that *StSN2* significantly affects the expression of *StBIN2*, which is a key negative regulator of the BR signaling pathway [38]. In the current study, we found that the transcript level and enzyme activity of StBIN2 were higher in *StSN2* overexpressing tubers (Fig. 1A–C). In addition, the transcript profiles of StSN2 and StBIN2 were similar during potato tuber storage and their expression levels were both high during the early stage of storage and decreased as storage period extended (Fig. 1D), and overexpression of *StBIN2* also resulted in extended tuber dormancy (Fig. 5B).

The Snakin/GASA proteins interact with other proteins to regulate various aspects of plant growth and development [12]. For example, the Arabidopsis AtGASA4 interacts with the receptor-like kinase (VHI/BRL2) to affect gibberellin (GA) signaling, thereby regulating the pattern of leaf venation [39]. Moreover, the rice OsGSR1 interacts with the BR biosynthetic enzyme DIM/DWF1 to regulate BR production [40]. Furthermore, the interaction between StSN2 and three peroxidases modulates lignin biosynthesis and H_2_O_2_ accumulation to inhibit tuber sprouting [16], and direct interaction between StSN2 and StGAPC1 also inhibits sprout growth [17]. In this study, we confirmed the interaction between StSN2 and StBIN2 by yeast two-hybrid, Co-IP, and LUC assays (Fig. 2A–C). The *in vitro* StBIN2 activity was the highest when it was in a 3:1 mass ratio with StSN2 compared with other StBIN2/StSN2 ratios and StBIN2 alone (Fig. 3B), suggesting that StSN2 interacts with StBIN2 to enhance its activity.

Bioinformatics analysis indicated that StSN2 and StBIN2 interact through multiple sites, among which cysteine residues are the most critical. We subsequently confirmed that substitution of these cysteine residues with alanine impaired the increase in StBIN2 activity when StSN2 was present (Fig. 4C). In eukaryotes, conserved cysteine sites are present in 9 of the 10 GSK3s in Arabidopsis. This high degree of conservation of GSK3 members is also present in humans and yeast [41]. Therefore, manipulation of BIN2 kinase activity has been achieved through mutagenesis of these cysteine residues [42]. In Arabidopsis, the kinase activity of AtBIN2 is inhibited through nitrosylation at cysteine residue 162 by *S*-nitrosoglutathione (GSNO) [43]. Cysteine has also been shown to possess mild redox activity [44]. For example, there are 12 cysteine residues in the GASA domain of Snakin/GASA proteins and these residues have been shown to influence their oxidation capacity [12]. Overexpression of *GASA5* in Arabidopsis has been reported to reduce the accumulation of reactive oxygen species under heat stress [45]. Similarly, in our previous study, we also found that *StSN2* could reduce the content of H_2_O_2_ in potato [16]. Treating the BIN2 protein with H_2_O_2_ promotes the oligomerization of BIN2 monomers, thus reducing its enzyme activity [46]. Recent studies have found that the enzymatic activity of StBIN2 is related to oxidation levels. In this study, we also detected a negative correlation between H_2_O_2_ content and StBIN2 activity in the budding eyes of tubers stored for 30 days (Supplementary Figs. S6 andS7). The dual luciferase complementary experiments further confirmed that cysteines in StBIN2 are critical for the binding of StSN2, as the increase in StBIN2 activity by StSN2 was impaired when the nine cysteine residues were replaced with alanine (Supplementary Fig. S8).

*Arabidopsis thaliana* BIN2 is one of the best-studied plant kinases, with critical roles in various signaling pathways including the BR and ABA pathways [47]. BZR1 is a key transcription factor in the BR signaling pathway [48]. BZR1 binds to the promoter of phytochrome-interacting factor 4 (PIF4) to induce PIF4 expression and PIF4 can activate growth-promoting genes to regulate cell elongation in Arabidopsis [49]. The *SUN* gene is a key regulator of fruit elongation in tomato and the binding of BZR1 to the E-box of SUN promoter activates *SUN* expression [50]. However, BIN2 phosphorylates BZR1 and inhibits its entry into the nucleus, suppressing the expression of BR-responsive genes and BR signaling. BIN2 also phosphorylates the core components of ABA signaling, such as SnRK2s and ABI5, to initiate ABA signal transduction. In Arabidopsis, BIN2 has been reported to phosphorylate and stabilize ABI5, thereby repressing seed germination [51]. AtBIN2 promotes ABA signaling by phosphorylating SnRK2.2 and SnRK2.3 [21]. In addition, BIN2 regulates the stability of vacuoleless gametophytes (VLG) proteins by interacting with and phosphorylating VLG, thus affecting the formation of large vacuoles in female gametophytes of Arabidopsis [52]. As described above, BIN2 is a protein kinase that coordinates plant growth and development [47]. Currently, there is limited research on the function of StBIN2 in potatoes. In our study, we found that overexpression of *StBIN2* maintained tuber dormancy (Fig. 5B). ABA and BR are both important hormones that regulate tuber dormancy and BIN2 is known to interact with SnRK2s, ABI5, and other factors in the ABA pathway to promote ABA signaling [51]. We investigated whether *StBIN2* influence potato dormancy by regulating ABA/BR signaling and found that *StBIN2* indeed enhances ABA signaling by promoting the expression of *SnRK2.2/2.3/2.4/2.6* and *ABI5* while suppressing BR signaling by inhibiting the expression *BZR1* (Fig. 6). Consistent with this result, the expression level of *NCED* in *StBIN2* overexpression lines increased whereas that of *DWF* decreased compared with WT (Supplementary Fig. S9). In addition, changes in ABA and BR levels during the same period are also consistent with those of *NCED* and *DWF* expression levels (Supplementary Fig. S10). These data support the notion that StBIN2 maintains tuber dormancy by affecting ABA/BR signaling.

Protein phosphorylation serves as a ubiquitous regulatory mechanism in the transmission of cellular signals [53]. The protein kinase BIN2 regulates downstream signaling molecules through phosphorylation and dephosphorylation [54]. For example, BIN2 has been shown to phosphorylate SnRK2.3 at T180 to enhance the kinase activity of SnRK2.3 in *A. thaliana* [21]. In this study, we confirmed the interaction between StBIN2 and StSnRK2.3 by yeast two-hybrid and luciferase complementation experiments. A band that represents the phosphorylated form of StSnRK2.3 was detected in *StBIN2* overexpression lines but not in the WT. Future studies are required to determine whether BIN2 also phosphorylates other SnRK2s members. Altogether, our study substantiates the interaction between StSN2 and StBIN2 and provides evidence for a role of this interaction in affecting ABA/BR signaling to maintain tuber dormancy (Fig. 8). Findings emerged from this significantly deepens our current understanding of the mechanism by which StSN2 maintains potato tuber dormancy through interaction with StSIN2 and provide guidance for the improvement of new varieties.

## Materials and methods

### Plant materials and postharvest storage

The full-length coding sequence of *StSN2* (Soltu.DM.01G050660.1) and *StBIN2* (Soltu.DM.03G001350.2) were subcloned into the *pBI121* binary vector driven by 35S cauliflower mosaic virus promoter. Similarly, the antisense sequences of *StSN2* was cloned into *pBI121* vector to create RNAi materials. Subsequently, *Agrobacterium tumefaciens* strain GV3101 carrying the recombinant constructs were cultured in *Agrobacterium rhizogenes* liquid medium (YEB) and shaken at 28°C overnight. On the following day, OD600 of the culture was adjusted to 0.6 with bacterial suspension (10 mM MES (pH 5.6), 10 mM MgCl_2_, and 0.2 mM acetosyringone). The stem of ‘Chuanyu 10’ (WT) tissue-cultured seedlings were cut into 0.5–1.0 cm length stem segments and immersed in the bacterial suspension for 5–8 min, cocultured at 28°C in dark for 36 h, and transferred to differentiation medium to generate adventitious buds. Gene transformation was carried out as described previously [23].

The sterile seedlings were propagated by plant tissue culture and kept at a temperature of 20 ± 2°C and light intensity of 100 μmol m^−2^ s^−1^, with 16 h light and 8 h dark. The tissue-cultured seedlings were grown for approximately 20 days on the MS medium and then transplanted into a seed bed containing peat soil to grow for 90 days prior to harvest. The harvested tubers were cleaned and placed under scattered light for wound healing. One week later, tubers of the same size were selected and stored at 20 ± 2°C away from direct light for subsequent study.

### Quantitative real-time PCR assays

Total RNA was extracted from the budding eyes of potato tubers using the Steady Pure Plant RNA Extraction Kit. cDNA was obtained using the Evo M-MLV RT Kit. Quantitative real-time PCR (qRT-PCR) was performed using the SYBR Green Premix Pro Taq HS qPCR Kit. The kits and reagents listed above were purchased from Accurate Biotechnology (Hunan, China). qRT-PCR data was obtained on the 7500 Real-Time PCR system. The 2^−ΔΔCt^ method was used to evaluate the relative transcription level of gene [24]. The elongation factor 1α-like (*EF1αL*) gene was used as the internal reference, and the primer sequences are listed in Supplementary Table S2. Three technical replicates were performed for these experiments.

### *In vitro* StBIN2 activity assay

The activity of StBIN2 in potato was assessed using the plant BIN2 ELISA Kit (Kexing, Shanghai, China). Firstly, 100 mg of budding eyes tissue of potato tubers was extracted and sample would be homogenized in grinders in conjunction. Forty microliters of diluent buffer and 10 μL of sample were added to each well. Then, 100 μL of HRP-conjugate reagent was added, and the plates were incubated for 60 min at 37°C. The reaction solution in the each well was discarded and the samples were washed five times with wash buffer. Fifty microliters of chromogen solution A and B was added to each well. The plates were incubated for another 15 min at 37°C in the dark. Finally, 50 μL of stop solution were added and the plates were placed in a microplate reader (Thermo Fisher Scientific, Massachusetts, USA) and measured for readouts at 450 nm. One hundred milligrams of sample consists of three potato budding eyes tissues and three biological replicates was performed for these experiments.

### Western blotting and co-immunoprecipitation assays

For the western blotting assay, anti-StSN2, StBIN2, and StSnRK2.3 antibodies were prepared in rabbits. Anti-tubin antibody was purchased from Servicebio (Wuhan, China). First, 30 μg of total protein from the budding eyes of three potato tubers was extracted. Then, proteins were separated with 10% sodium dodecyl sulfate–polyacrylamide gel electrophoresis (SDS-PAGE) and transferred to the nitrocellulose membrane using the wet transfer method [25]. The nitrocellulose membrane was incubated with respective antibodies, and the target proteins were detected using the BeyoECL Plus Kit (Beyotime, China) [26].

Co-IP assays were performed as described previously [27]. The total protein was extracted from the budding eyes of potato tubers and transferred to a centrifuge tube, and 10 μL of purified anti-StBIN2 antibody and 20 μL protein A magnetic beads (Beyotime, China) were sequentially added to the centrifuge tube. The centrifuge tube was fixed on a rotating shaking bed at 10 rpm/min for 6–8 h, at 4°C, and then centrifuged at 3000×*g* for 5 min. The supernatant was discarded, and the solution was then precipitated with 1× PBS three times. Then, 30 μL of loading buffer was added and the sample was placed in a boiling water bath for 10 min. The sample was then centrifuged for 1 min, and the supernatant was used for Co-IP detection.

### Yeast two-hybrid and luciferase complementation assays

For the yeast two-hybrid assay, the open reading frame (ORF) sequences of *StSnRK2.3* and *StSN2* were individually inserted into the *pGBKT7* vector, and the ORF of *StBIN2* was ligated into the *pGADT7* vector. The resulting *AD-StBIN2* and *BD-StSN2* constructs were transformed into AH109 yeast cells by the PEG/LiAc method [28]. *AD-BIN2* and *BD-StSnRK2.3* were transformed into AH109 yeast cells with the same method. After interaction, the yeast was grown on SD-Trp/Leu/His/Ade culture medium, with AtCBL1 and AtCIPK23 serving as positive controls [29] and *BD-StSN2* and AD-Empty as negative controls.

For the luciferase complementation assays, the ORFs of *StSnRK2.3* and *StSN2* were individually cloned into the *pCAMBIA-cLUC* vector and that of *StBIN2* was cloned into the *pCAMBIA-nLUC* vector. These vectors were separately transformed into the GV3101 strain and the bacterial solution was injected into the leaves of *Nicotiana benthamiana*. After growing for 3 days, luminescence was recorded using a CCD camera (Viber Fusion FX, France) and the luciferase activity was detected using a dual luciferase reporter assay kit (Vazyme, China) [30]. The primers are listed in Supplementary Table S2.

### *In vitro* kinase assay

In order to obtain StSN2 and StBIN2 proteins, the ORFs of *StSN2* and *StBIN2* were inserted into the *Pcold-TF* vector, and the recombinant plasmid was transformed into BL21 *Escherichia coli*. Expression of the fusion protein was induced at 16°C by adding isopropyl β-d-thiogalactoside (IPTG) to the culture medium to a final concentration of 1 mM. The Ni IDA column (Smart-Life Science, China) was used for protein purification. Five micrograms of purified StBIN2 protein for enzyme activity detection and BIN2 kinase activity was assayed using the Kinase-Lumi Plus Luminescent Kinase Assay Kit (Beyotime, China). The kinase activity was calculated as described previously [31]. Three biological replicates was performed for these experiments.

### Site-directed mutagenesis of *StBIN2*

To confirm the potential cysteine interaction sites between StSN2 and StBIN2, we used the amino acid sequences as inputs in the HDOCK server (http://hdock.phys.hust.edu.cn/) [32] and searched for potential interaction sites between StSN2 and StBIN2 through homology searching, template modeling, structural prediction, macromolecular docking, biological information incorporation, fast protein–protein docking homology modeling, and macromolecular docking. Mutation of the interaction sites between StSN2 and StBIN2 were generated by gene synthesis.

### Phos-tag mobility shift assay

The Phos-tag mobility shift assay was carried out as described previously [33]. Total protein was extracted from potato budding eyes tissue and separated in a 10% SDS-PAGE that contained 50 mM Phos-tag. After electrophoresis, the gel was washed three times in transfer buffer (50 mM Tris, 40 mM glycine) for 10 min per wash. Then, the gel was transferred to a polyvinylidene fluoride membrane. The polyvinylidene fluoride membrane was incubated with the StSnRK2.3 antibody and the protein was detected using the BeyoECL Plus Kit (Beyotime, China) [26].

### Statistical analysis

All experiments were performed in triplicate and the data are shown as mean ± SE (*n* = 3). Significant differences were determined using the Student’s *t*-test. Different letters in the figures represent significant differences among samples at *P* ≤ 0.01 and *P* ≤ 0.05 levels. SPSS and Origin 2021 software were used to analyze the data.

## Supplementary Material

Web_Material_uhad228

## Data Availability

All relevant data can be found within the manuscript and its supporting materials.
