# Supplementary material for: StSN2 interacts with the brassinosteroid signaling suppressor StBIN2 to maintain tuber dormancy
Source: Hortic Res. 2023 Nov 8;10(12):uhad228. doi: 10.1093/hr/uhad228 (PMC10753161; doi:10.1093/hr/uhad228)
Supplement: Web_Material_uhad228 [file web_material_uhad228.zip › Supplementary Table S1.pdf]

Supplementary 1. The identified StBIN2 from CoIP-MS

Accession	Protein names	Gene names	MW [kDa]	Prorein score in OEX27	Prorein Coverage (%)	Number Of Unique Peptides	Number Of Peptides	Number Of Matched spectrum
M1CJG6	Protein kinase domain-containing protein	-	41.41	95.80	6.08	2	2	2
