# Supplementary material for: StSN2 interacts with the brassinosteroid signaling suppressor StBIN2 to maintain tuber dormancy
Source: Hortic Res. 2023 Nov 8;10(12):uhad228. doi: 10.1093/hr/uhad228 (PMC10753161; doi:10.1093/hr/uhad228)
Supplement: Web_Material_uhad228 [file web_material_uhad228.zip › Supplementary Table S2.pdf]

Table S2. Quantitative RT-PCR for RNA sequencing validation.

Gene	Purpose	Forward/Reverse
StSN2	Cloning of OE	GGATCCATGGCCATTTCGAAAGCTCT
		GGATCCATGGCCATTTCGAAAGCTCT
StSN2	Cloning of RNAi	CCCCGGATGGCCATTTCGAAAGCTCT
		GGATCCTTAAGGGCATTTACGTTTGT
StSN2	qPCR	TAACAGATGTAGCCACTGAC
		ACAACAAGTTCCACATGCCC
StSN2	Yeast two-kybrid	CAGAGGAGGACCTGCATATGGCCATTTCGAAAGCTCT
		GCAGGTCGACGGATCCAGGGCATTTACGTTTGT
StSN2	Luciferase	CGCGTCCCGGGGCGGTACATATGGCCATTTCGAAAGCTCT
	complementary	TCCTTGTAGTCCATTTGTTGGATCCAGGGCATTTACGTTTGT
StSN2	kKinase assay	GGAGCTCGGTACCCTCGAGGGATCCATGGCCATTTCGAAAGC
		TTAAGCAGAGATTACCTATCTAGATTAAGGGCATTTACGTTTGT
StBIN2	Cloning of OE	GGATCCATGGCTGAACGTATTGTGGG
		GGATCCTTATGCCAAGGGTGTGCTT
StBIN2	qPCR	CTGCTCACGGTTTTATCGGG
		TCCTCTCTCGTTGGTGTTCC
StBIN2	kinase assay	CGCGGATCCATGGCTGATGATAAGGAGATGTCT
		CTAGTCTAGATCACGTCATGTCATCACGGG
StSnRK2.3	luciferase	GGAGCTCGGTACCCTCGAGGGATGGATCGGACGGCAGTGA
	complementary	TTAAGCAGAGATTACCTATCTAGATTACATTGCATAGACAATCTCT
StSnRK2.3	Yeast two-kybrid	CCAGATTACGCTCATATGATGGATCGGACGGCAGT
		GTGGAATTC TTACATTGCATAGACAATCTCTC
StSnRK2.2	qPCR	TTATGGAGTACGCAGCAGGT
		CCCACAGTCGACTTTGGTTG
StSnRK2.3	qPCR	TGTATGTCATGCTGGTGGGT
		ACGTTGATCTGGCTCCTCAA
StSnRK2.4	qPCR	TTTTGGCCTCGAATGCAACA
		ACGTTGATCTGGCTCCTCAA
StSnRK2.6	qPCR	GATCGCATCTGTCAAGCTGG
		ACTTGGGACGTGAATGCAAC
