# Supplementary material for: StSN2 interacts with the brassinosteroid signaling suppressor StBIN2 to maintain tuber dormancy
Source: Hortic Res. 2023 Nov 8;10(12):uhad228. doi: 10.1093/hr/uhad228 (PMC10753161; doi:10.1093/hr/uhad228)
Supplement: Web_Material_uhad228 [file web_material_uhad228.zip › Supplementary fig 4.pdf]

MADDKEMSAPVMDVNGGVTGHIISTTIGGKNGEPKQTVSYMAERIVGTGSFGVVFQAKCLENGETVAIKKVLQDRRYKNRELQLMRTMDNPNVVSLKHCFYSTTSKNELF
1 10 20 30 40 50 60 70 80 90 100 110

LNLVMEYVPETMYRMLKHYSNMNQRMPLIYVKLYTYQVFRGLAYMHTVADVCHRDLKPQNILVDPVTHQVKICDFGSAKVLVKGEANISYICSRFYRAPELIFGATEYTT
120 130 140 150 160 170 180 190 200 210 220

SIDIWSAGCVLAELLLGQPLFPGENAVDQLVEIIKVLGTPTREEIRCMNPNYTDFRFPQIKAHPWHKVFHKRMPPEAIDLASRLLQYSPSLRCNALEACAHPFFDELREP
230 240 250 260 270 280 290 300 310 320 330

NARLPHGRQLPPLFNFKQELAGASPDLINRLIPDHIKRQMGLHHFTTRDDMT
340 350 360 370 380 382
