# Supplementary figures and images for: StSN2 interacts with the brassinosteroid signaling suppressor StBIN2 to maintain tuber dormancy
Source: Hortic Res. 2023 Nov 8;10(12):uhad228. doi: 10.1093/hr/uhad228 (PMC10753161; doi:10.1093/hr/uhad228)

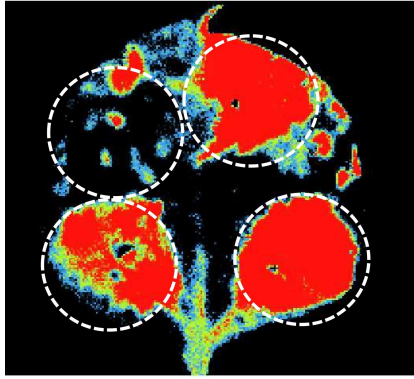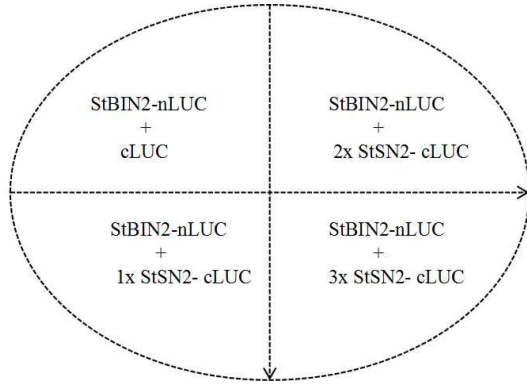

Supplement: Web_Material_uhad228 [file web_material_uhad228.zip › Supplementary fig 2.pdf]

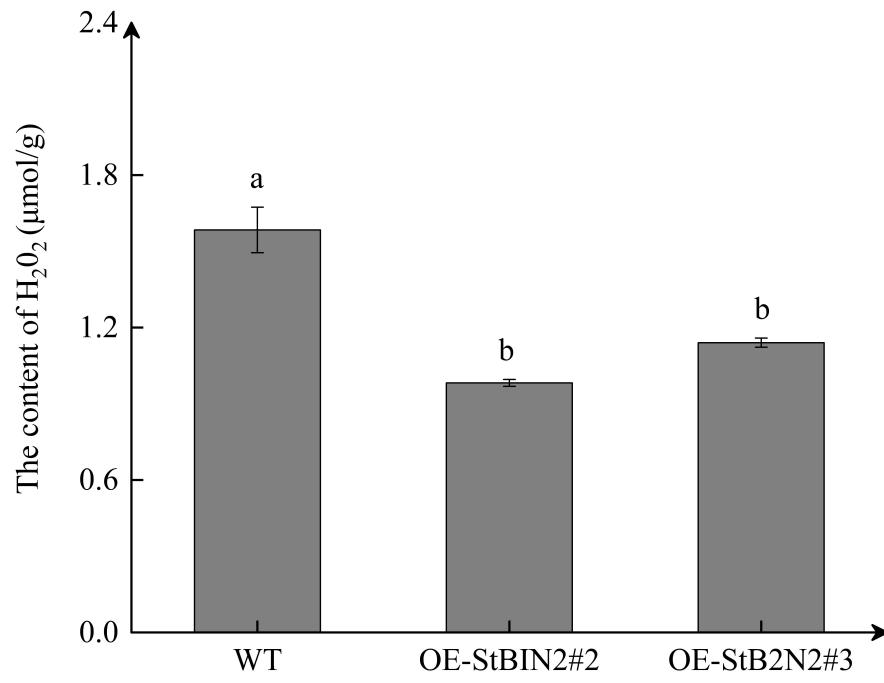

Supplement: Web_Material_uhad228 [file web_material_uhad228.zip › Supplementary fig 7.pdf]

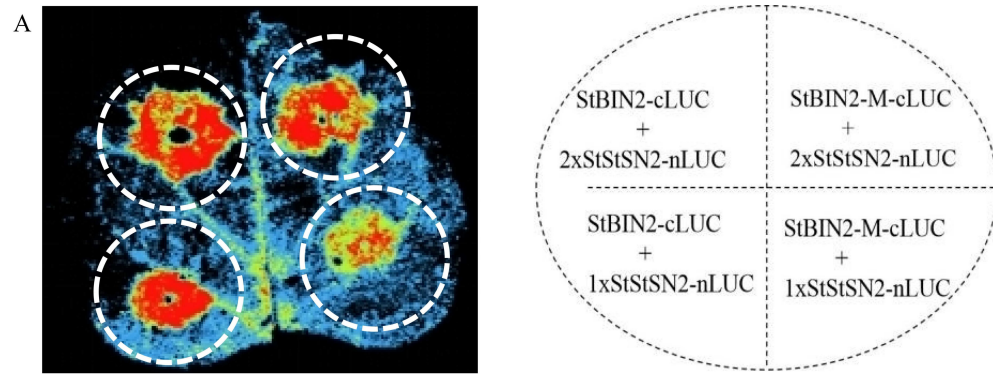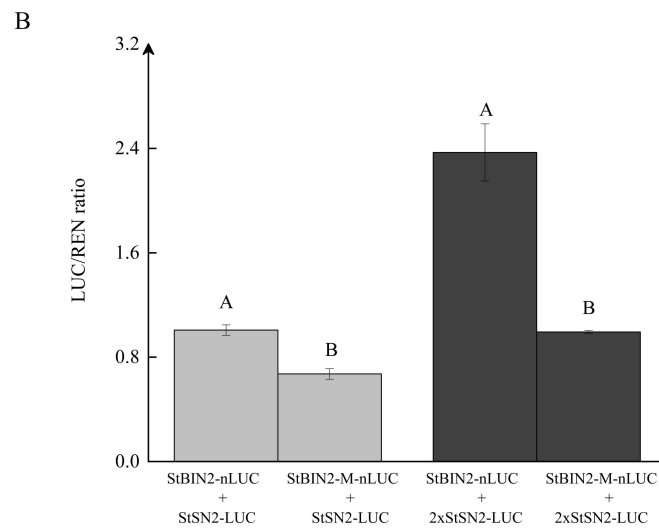

Supplement: Web_Material_uhad228 [file web_material_uhad228.zip › Supplementary fig 8.pdf]

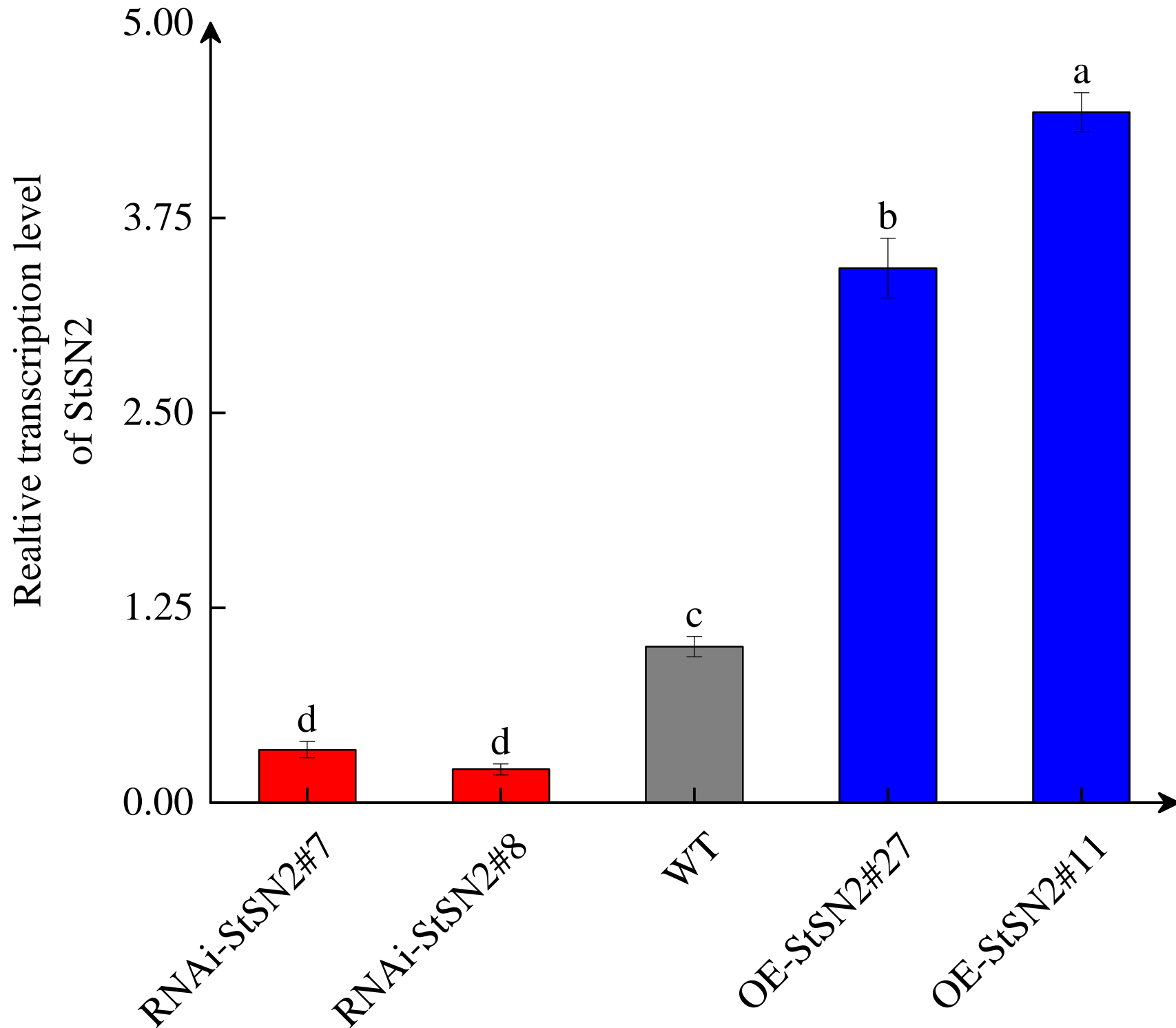

Supplement: Web_Material_uhad228 [file web_material_uhad228.zip › Supplementary fig 1.pdf]

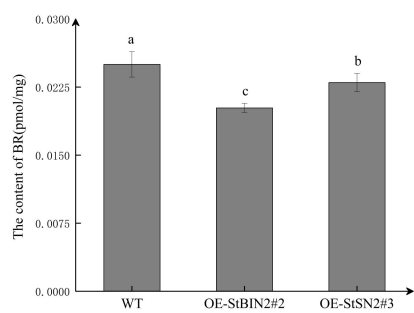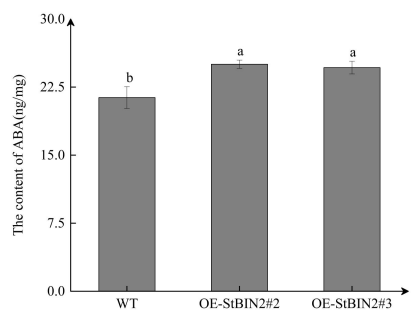

Supplement: Web_Material_uhad228 [file web_material_uhad228.zip › Supplementary fig 10.pdf]

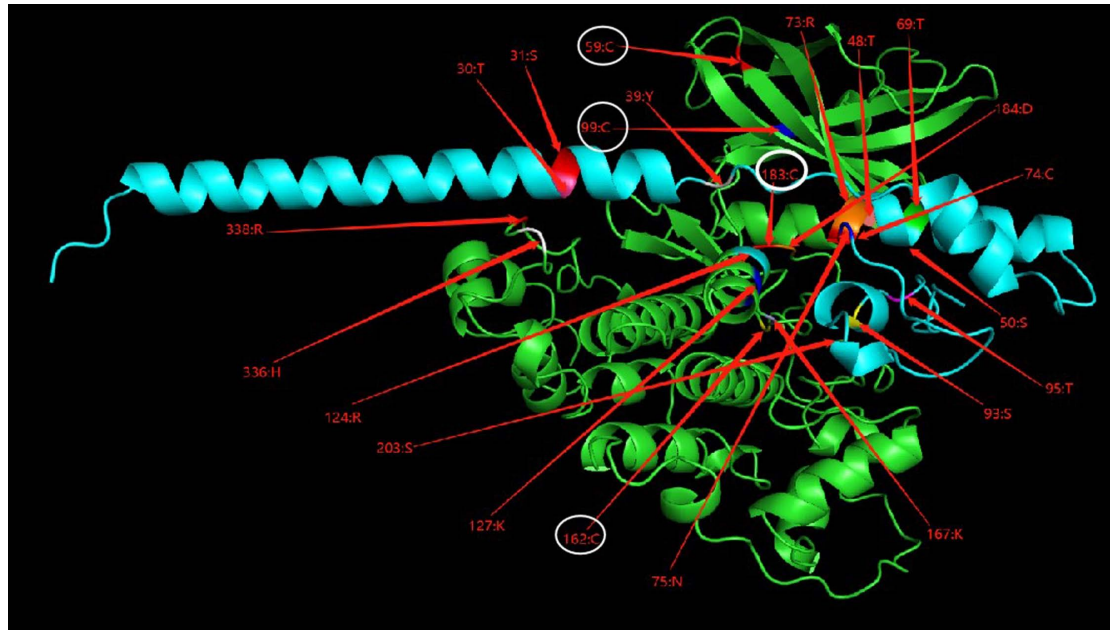

Supplement: Web_Material_uhad228 [file web_material_uhad228.zip › Supplementary fig 3.pdf]

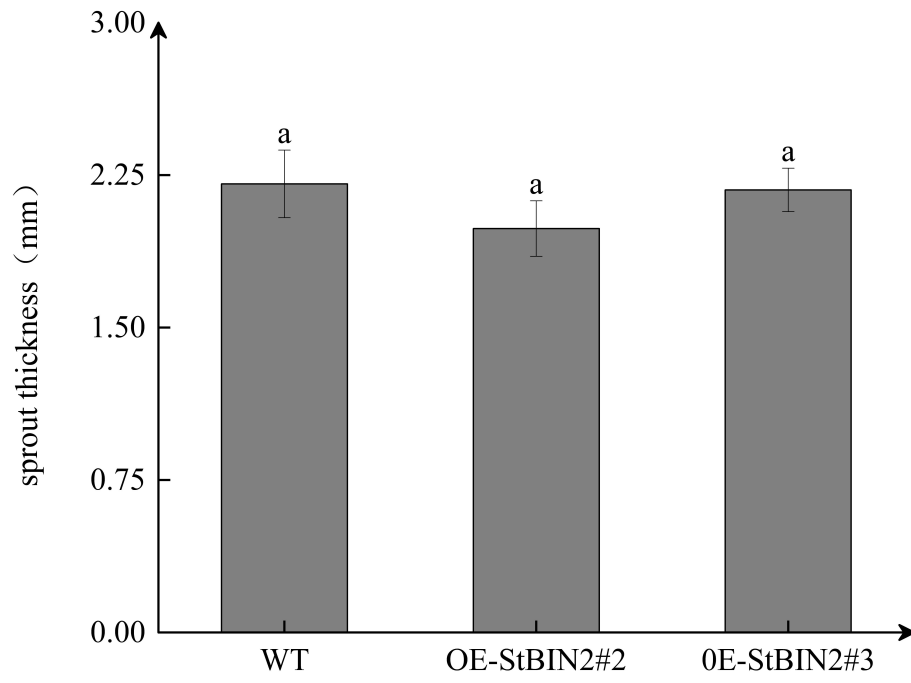

Supplement: Web_Material_uhad228 [file web_material_uhad228.zip › Supplementary fig 5.pdf]

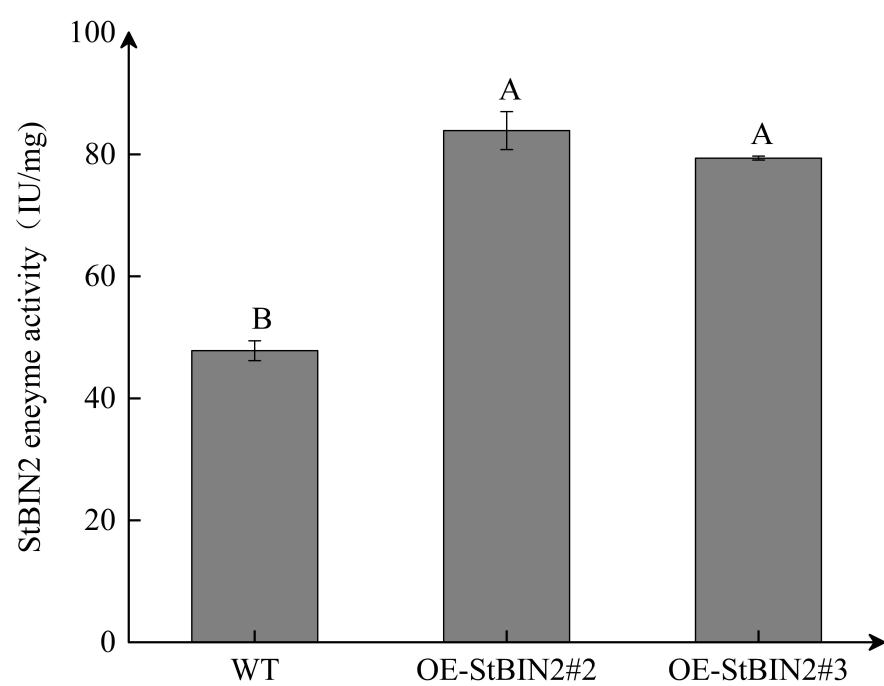

Supplement: Web_Material_uhad228 [file web_material_uhad228.zip › Supplementary fig 6.pdf]

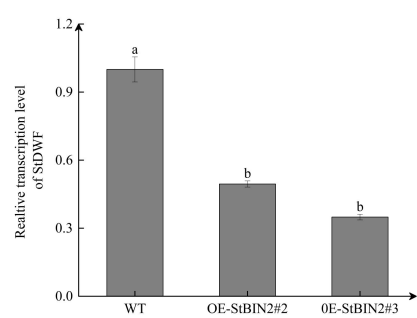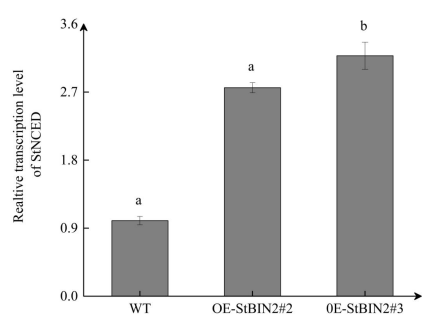

Supplement: Web_Material_uhad228 [file web_material_uhad228.zip › Supplementary fig 9.pdf]
